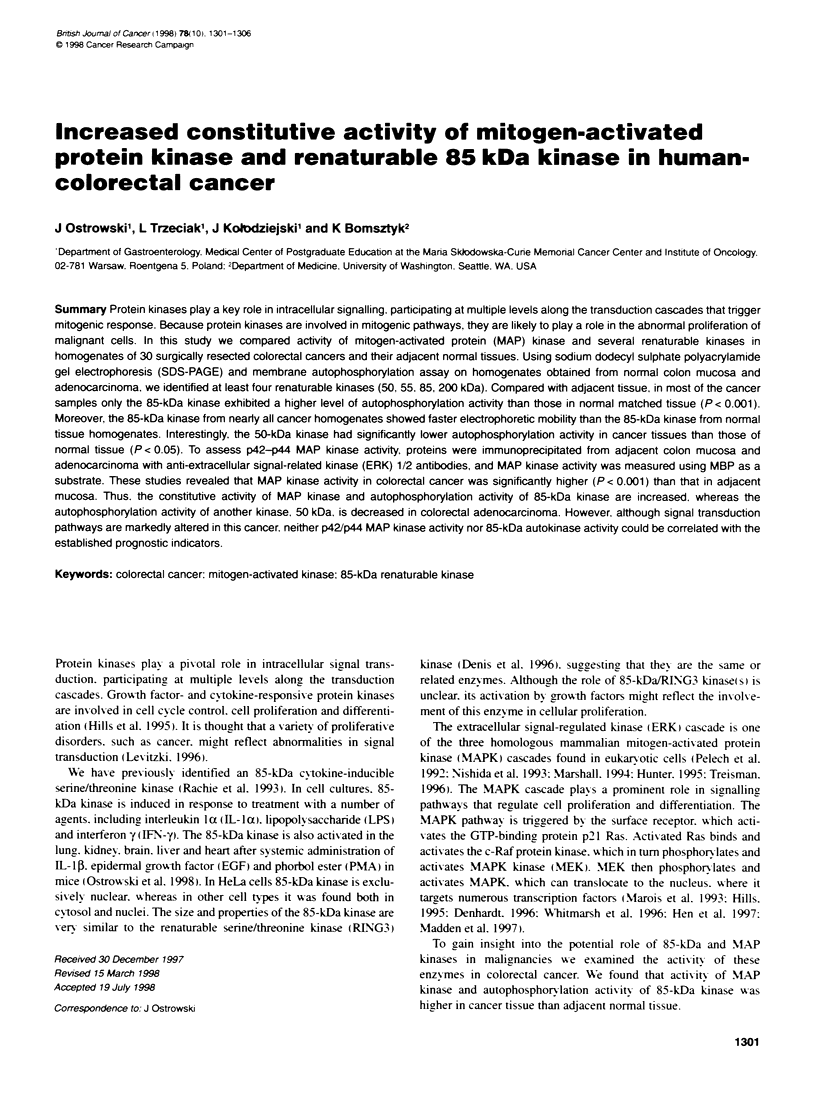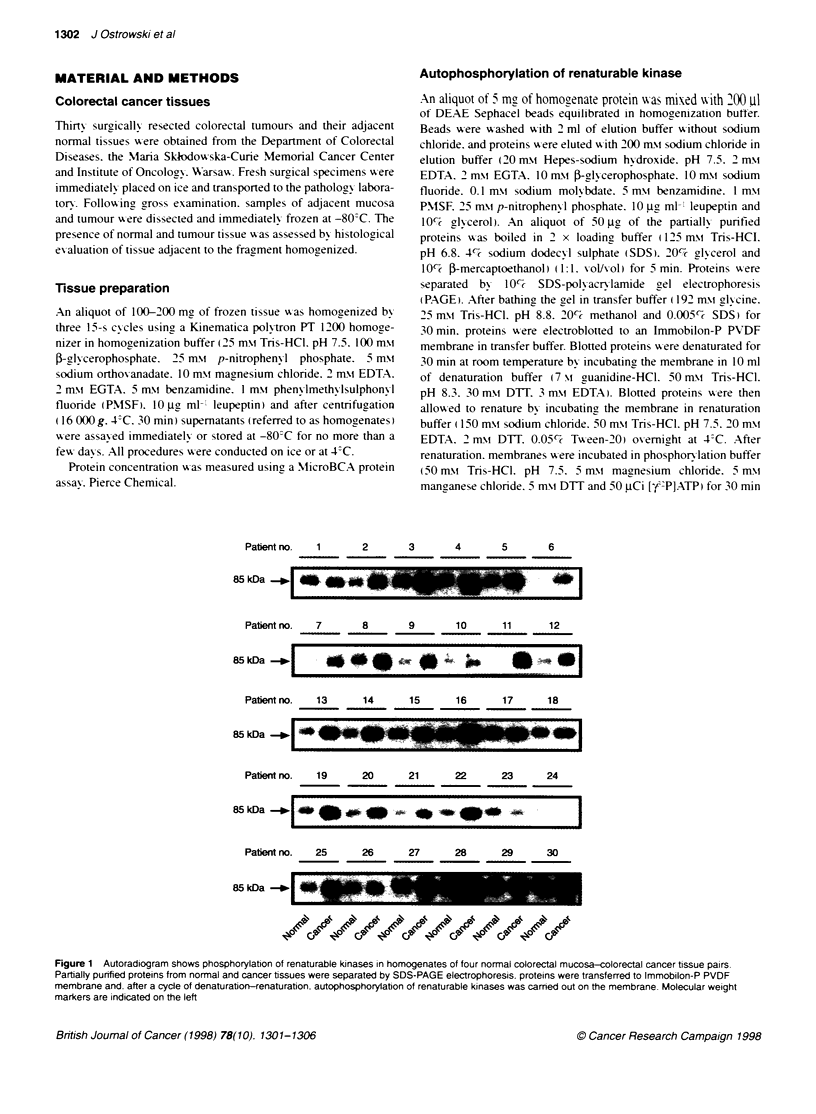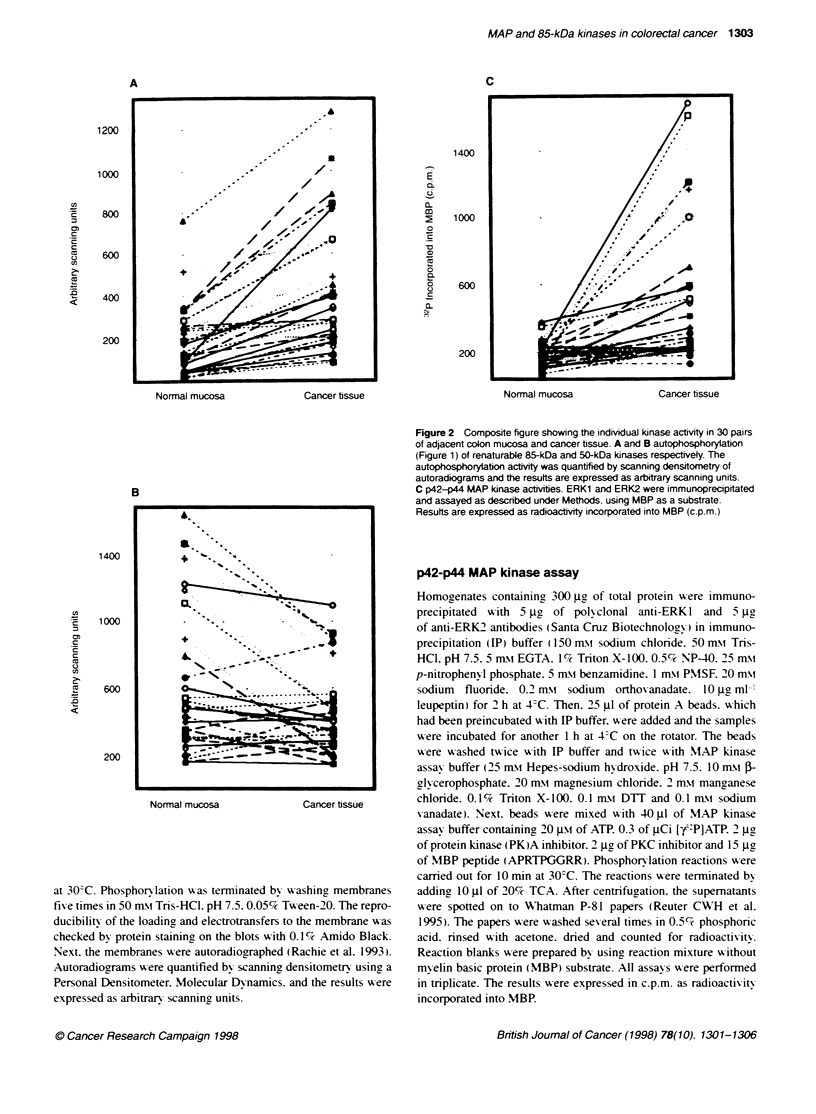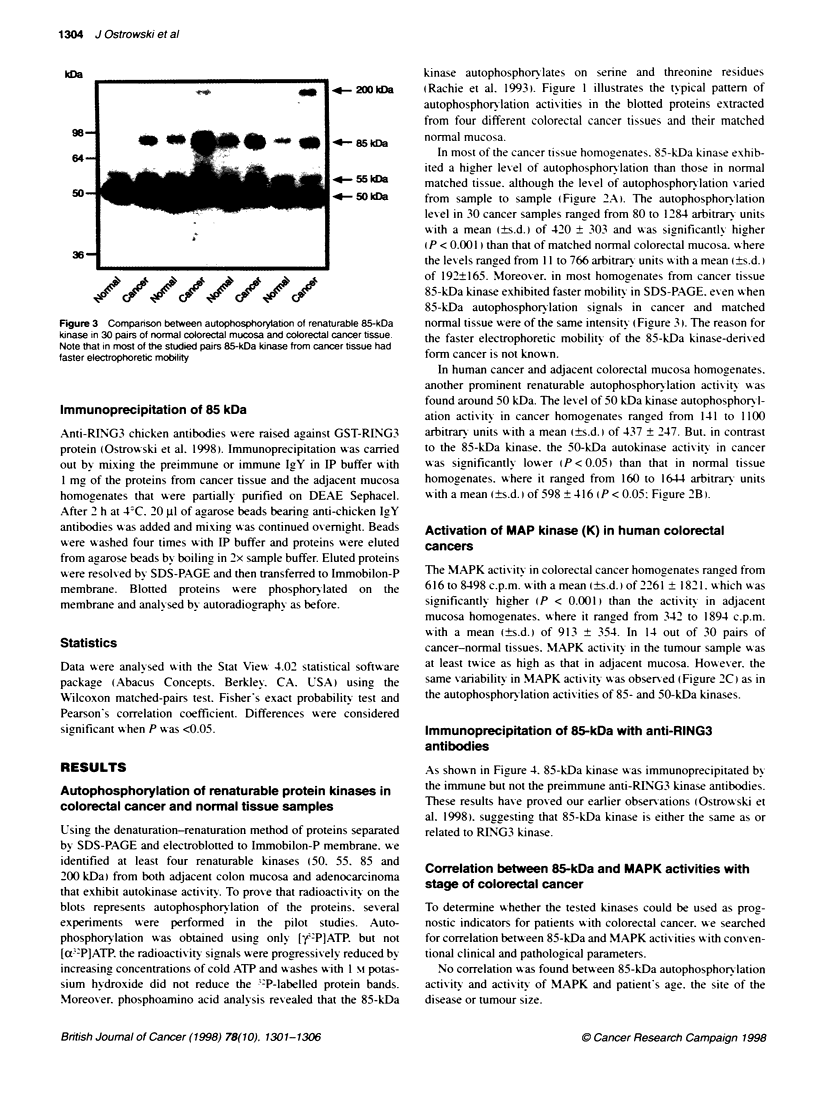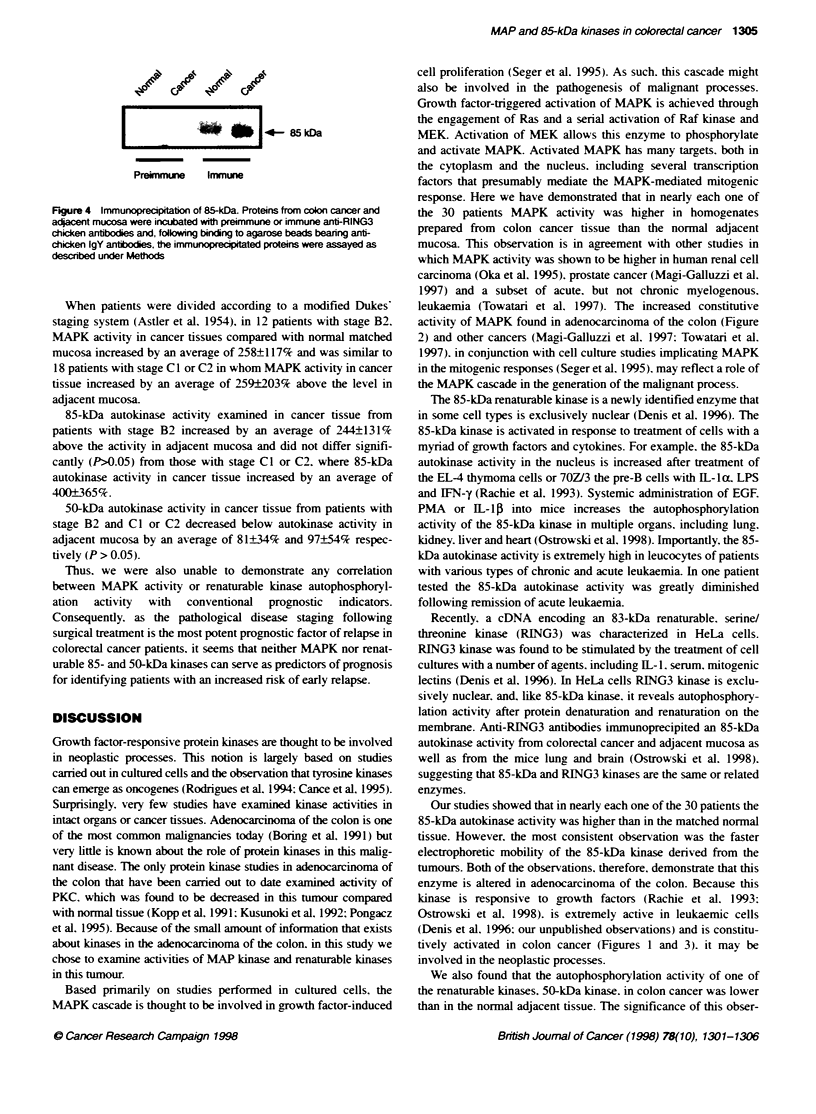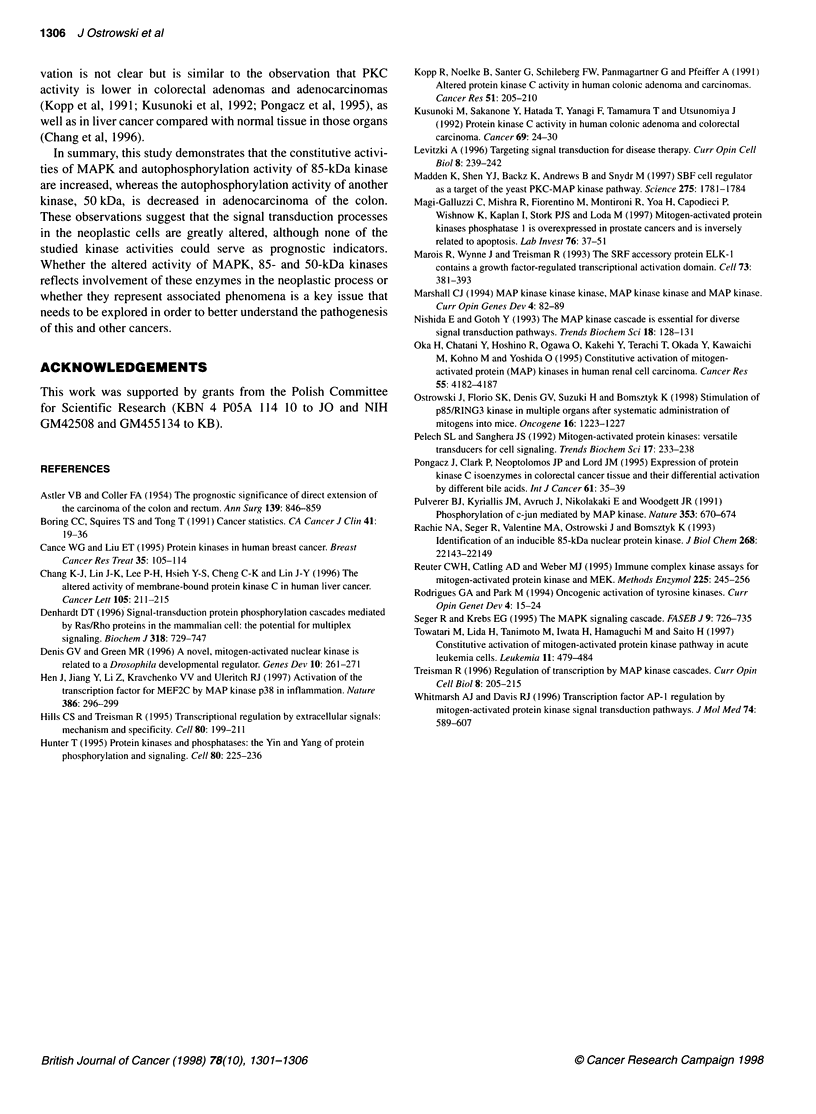# Increased constitutive activity of mitogen-activated protein kinase and renaturable 85 kDa kinase in human-colorectal cancer.

**DOI:** 10.1038/bjc.1998.675

**Published:** 1998-11

**Authors:** J. Ostrowski, L. Trzeciak, J. Kołodziejski, K. Bomsztyk

**Affiliations:** Department of Gastroenterology, Medical Center of Postgraduate Education at the Maria Skłodowska-Curie Memorial Cancer Center and Institute of Oncology, Warsaw, Poland.

## Abstract

**Images:**


British Journal of Cancer (1998) 78(10), 1301–1306
© 1998 Cancer Research Campaign

### Increased constitutive activity of mitogen-activated protein kinase and renaturable 85 kDa kinase in human colorectal cancer

**J Ostrowski[1], L Trzeciak[1], J Kołodziejski[1] and K Bomsztyk[2]**

[1]Department of Gastroenterology, Medical Center of Postgraduate Education at the Maria Skłodowska-Curie Memorial Cancer Center and Institute of Oncology, 02-781 Warsaw, Roentgena 5, Poland; [2]Department of Medicine, University of Washington, Seattle, WA, USA

**Summary** Protein kinases play a key role in intracellular signalling, participating at multiple levels along the transduction cascades that trigger mitogenic response. Because protein kinases are involved in mitogenic pathways, they are likely to play a role in the abnormal proliferation of malignant cells. In this study we compared activity of mitogen-activated protein (MAP) kinase and several renaturable kinases in homogenates of 30 surgically resected colorectal cancers and their adjacent normal tissues. Using sodium dodecyl sulphate polyacrylamide gel electrophoresis (SDS-PAGE) and membrane autophosphorylation assay on homogenates obtained from normal colon mucosa and adenocarcinoma, we identified at least four renaturable kinases (50, 55, 85, 200 kDa). Compared with adjacent tissue, in most of the cancer samples only the 85-kDa kinase exhibited a higher level of autophosphorylation activity than those in normal matched tissue ($P < 0.001$). Moreover, the 85-kDa kinase from nearly all cancer homogenates showed faster electrophoretic mobility than the 85-kDa kinase from normal tissue homogenates. Interestingly, the 50-kDa kinase had significantly lower autophosphorylation activity in cancer tissues than those of normal tissue ($P < 0.05$). To assess p42–p44 MAP kinase activity, proteins were immunoprecipitated from adjacent colon mucosa and adenocarcinoma with anti-extracellular signal-related kinase (ERK) 1/2 antibodies, and MAP kinase activity was measured using MBP as a substrate. These studies revealed that MAP kinase activity in colorectal cancer was significantly higher ($P < 0.001$) than that in adjacent mucosa. Thus, the constitutive activity of MAP kinase and autophosphorylation activity of 85-kDa kinase are increased, whereas the autophosphorylation activity of another kinase, 50 kDa, is decreased in colorectal adenocarcinoma. However, although signal transduction pathways are markedly altered in this cancer, neither p42/p44 MAP kinase activity nor 85-kDa autokinase activity could be correlated with the established prognostic indicators.

**Keywords:** colorectal cancer; mitogen-activated kinase; 85-kDa renaturable kinase

Protein kinases play a pivotal role in intracellular signal transduction, participating at multiple levels along the transduction cascades. Growth factor- and cytokine-responsive protein kinases are involved in cell cycle control, cell proliferation and differentiation (Hills et al. 1995). It is thought that a variety of proliferative disorders, such as cancer, might reflect abnormalities in signal transduction (Levitzki, 1996).

We have previously identified an 85-kDa cytokine-inducible serine/threonine kinase (Rachie et al. 1993). In cell cultures, 85-kDa kinase is induced in response to treatment with a number of agents, including interleukin 1$\alpha$ (IL-1$\alpha$), lipopolysaccharide (LPS) and interferon $\gamma$ (IFN-$\gamma$). The 85-kDa kinase is also activated in the lung, kidney, brain, liver and heart after systemic administration of IL-1$\beta$, epidermal growth factor (EGF) and phorbol ester (PMA) in mice (Ostrowski et al. 1998). In HeLa cells 85-kDa kinase is exclusively nuclear, whereas in other cell types it was found both in cytosol and nuclei. The size and properties of the 85-kDa kinase are very similar to the renaturable serine/threonine kinase (RING3) kinase (Denis et al. 1996), suggesting that they are the same or related enzymes. Although the role of 85-kDa/RING3 kinase(s) is unclear, its activation by growth factors might reflect the involvement of this enzyme in cellular proliferation.

The extracellular signal-regulated kinase (ERK) cascade is one of the three homologous mammalian mitogen-activated protein kinase (MAPK) cascades found in eukaryotic cells (Pelech et al. 1992; Nishida et al. 1993; Marshall, 1994; Hunter, 1995; Treisman, 1996). The MAPK cascade plays a prominent role in signalling pathways that regulate cell proliferation and differentiation. The MAPK pathway is triggered by the surface receptor, which activates the GTP-binding protein p21 Ras. Activated Ras binds and activates the c-Raf protein kinase, which in turn phosphorylates and activates MAPK kinase (MEK). MEK then phosphorylates and activates MAPK, which can translocate to the nucleus, where it targets numerous transcription factors (Marois et al. 1993; Hills, 1995; Denhardt, 1996; Whitmarsh et al. 1996; Hen et al. 1997; Madden et al. 1997).

To gain insight into the potential role of 85-kDa and MAP kinases in malignancies we examined the activity of these enzymes in colorectal cancer. We found that activity of MAP kinase and autophosphorylation activity of 85-kDa kinase was higher in cancer tissue than adjacent normal tissue.

*Received 30 December 1997*
*Revised 15 March 1998*
*Accepted 19 July 1998*

*Correspondence to:* J Ostrowski

### MATERIAL AND METHODS

### Colorectal cancer tissues

Thirty surgically resected colorectal tumours and their adjacent normal tissues were obtained from the Department of Colorectal Diseases, the Maria Skłodowska-Curie Memorial Cancer Center and Institute of Oncology, Warsaw. Fresh surgical specimens were immediately placed on ice and transported to the pathology laboratory. Following gross examination, samples of adjacent mucosa and tumour were dissected and immediately frozen at −80°C. The presence of normal and tumour tissue was assessed by histological evaluation of tissue adjacent to the fragment homogenized.

### Tissue preparation

An aliquot of 100–200 mg of frozen tissue was homogenized by three 15-s cycles using a Kinematica polytron PT 1200 homogenizer in homogenization buffer (25 mM Tris-HCl, pH 7.5, 100 mM β-glycerophosphate, 25 mM *p*-nitrophenyl phosphate, 5 mM sodium orthovanadate, 10 mM magnesium chloride, 2 mM EDTA, 2 mM EGTA, 5 mM benzamidine, 1 mM phenylmethylsulphonyl fluoride (PMSF), 10 µg $ml^{-1}$ leupeptin) and after centrifugation (16 000 ***g***, 4°C, 30 min) supernatants (referred to as homogenates) were assayed immediately or stored at −80°C for no more than a few days. All procedures were conducted on ice or at 4°C.

Protein concentration was measured using a MicroBCA protein assay, Pierce Chemical.

### Autophosphorylation of renaturable kinase

An aliquot of 5 mg of homogenate protein was mixed with 200 µl of DEAE Sephacel beads equilibrated in homogenization buffer. Beads were washed with 2 ml of elution buffer without sodium chloride, and proteins were eluted with 200 mM sodium chloride in elution buffer (20 mM Hepes-sodium hydroxide, pH 7.5, 2 mM EDTA, 2 mM EGTA, 10 mM β-glycerophosphate, 10 mM sodium fluoride, 0.1 mM sodium molybdate, 5 mM benzamidine, 1 mM PMSF, 25 mM *p*-nitrophenyl phosphate, 10 µg $ml^{-1}$ leupeptin and 10% glycerol). An aliquot of 50 µg of the partially purified proteins was boiled in 2 × loading buffer (125 mM Tris-HCl, pH 6.8, 4% sodium dodecyl sulphate (SDS), 20% glycerol and 10% β-mercaptoethanol) (1:1, vol/vol) for 5 min. Proteins were separated by 10% SDS-polyacrylamide gel electrophoresis (PAGE). After bathing the gel in transfer buffer (192 mM glycine, 25 mM Tris-HCl, pH 8.8, 20% methanol and 0.005% SDS) for 30 min, proteins were electroblotted to an Immobilon-P PVDF membrane in transfer buffer. Blotted proteins were denaturated for 30 min at room temperature by incubating the membrane in 10 ml of denaturation buffer (7 M guanidine-HCl, 50 mM Tris-HCl, pH 8.3, 30 mM DTT, 3 mM EDTA). Blotted proteins were then allowed to renature by incubating the membrane in renaturation buffer (150 mM sodium chloride, 50 mM Tris-HCl, pH 7.5, 20 mM EDTA, 2 mM DTT, 0.05% Tween-20) overnight at 4°C. After renaturation, membranes were incubated in phosphorylation buffer (50 mM Tris-HCl, pH 7.5, 5 mM magnesium chloride, 5 mM manganese chloride, 5 mM DTT and 50 µCi $[\gamma^{32}P]$ATP) for 30 min

**Figure 1** Autoradiogram shows phosphorylation of renaturable kinases in homogenates of four normal colorectal mucosa–colorectal cancer tissue pairs. Partially purified proteins from normal and cancer tissues were separated by SDS-PAGE electrophoresis, proteins were transferred to Immobilon-P PVDF membrane and, after a cycle of denaturation–renaturation, autophosphorylation of renaturable kinases was carried out on the membrane. Molecular weight markers are indicated on the left

© Cancer Research Campaign 1998

**Figure 2** Composite figure showing the individual kinase activity in 30 pairs of adjacent colon mucosa and cancer tissue. **A** and **B** autophosphorylation (Figure 1) of renaturable 85-kDa and 50-kDa kinases respectively. The autophosphorylation activity was quantified by scanning densitometry of autoradiograms and the results are expressed as arbitrary scanning units. **C** p42–p44 MAP kinase activities. ERK1 and ERK2 were immunoprecipitated and assayed as described under Methods, using MBP as a substrate. Results are expressed as radioactivity incorporated into MBP (c.p.m.)

at 30°C. Phosphorylation was terminated by washing membranes five times in 50 mM Tris-HCl, pH 7.5, 0.05% Tween-20. The reproducibility of the loading and electrotransfers to the membrane was checked by protein staining on the blots with 0.1% Amido Black. Next, the membranes were autoradiographed (Rachie et al, 1993). Autoradiograms were quantified by scanning densitometry using a Personal Densitometer, Molecular Dynamics, and the results were expressed as arbitrary scanning units.

### p42-p44 MAP kinase assay

Homogenates containing 300 μg of total protein were immunoprecipitated with 5 μg of polyclonal anti-ERK1 and 5 μg of anti-ERK2 antibodies (Santa Cruz Biotechnology) in immunoprecipitation (IP) buffer (150 mM sodium chloride, 50 mM Tris-HCl, pH 7.5, 5 mM EGTA, 1% Triton X-100, 0.5% NP-40, 25 mM *p*-nitrophenyl phosphate, 5 mM benzamidine, 1 mM PMSF, 20 mM sodium fluoride, 0.2 mM sodium orthovanadate, 10 μg ml$^{-1}$ leupeptin) for 2 h at 4°C. Then, 25 μl of protein A beads, which had been preincubated with IP buffer, were added and the samples were incubated for another 1 h at 4°C on the rotator. The beads were washed twice with IP buffer and twice with MAP kinase assay buffer (25 mM Hepes-sodium hydroxide, pH 7.5, 10 mM β-glycerophosphate, 20 mM magnesium chloride, 2 mM manganese chloride, 0.1% Triton X-100, 0.1 mM DTT and 0.1 mM sodium vanadate). Next, beads were mixed with 40 μl of MAP kinase assay buffer containing 20 μM of ATP, 0.3 of μCi [γ$^{32}$P]ATP, 2 μg of protein kinase (PK)A inhibitor, 2 μg of PKC inhibitor and 15 μg of MBP peptide (APRTPGGRR). Phosphorylation reactions were carried out for 10 min at 30°C. The reactions were terminated by adding 10 μl of 20% TCA. After centrifugation, the supernatants were spotted on to Whatman P-81 papers (Reuter CWH et al, 1995). The papers were washed several times in 0.5% phosphoric acid, rinsed with acetone, dried and counted for radioactivity. Reaction blanks were prepared by using reaction mixture without myelin basic protein (MBP) substrate. All assays were performed in triplicate. The results were expressed in c.p.m. as radioactivity incorporated into MBP.

**Figure 3** Comparison between autophosphorylation of renaturable 85-kDa kinase in 30 pairs of normal colorectal mucosa and colorectal cancer tissue. Note that in most of the studied pairs 85-kDa kinase from cancer tissue had faster electrophoretic mobility

### Immunoprecipitation of 85 kDa

Anti-RING3 chicken antibodies were raised against GST-RING3 protein (Ostrowski et al. 1998). Immunoprecipitation was carried out by mixing the preimmune or immune IgY in IP buffer with 1 mg of the proteins from cancer tissue and the adjacent mucosa homogenates that were partially purified on DEAE Sephacel. After 2 h at 4°C, 20 µl of agarose beads bearing anti-chicken IgY antibodies was added and mixing was continued overnight. Beads were washed four times with IP buffer and proteins were eluted from agarose beads by boiling in 2× sample buffer. Eluted proteins were resolved by SDS-PAGE and then transferred to Immobilon-P membrane. Blotted proteins were phosphorylated on the membrane and analysed by autoradiography as before.

### Statistics

Data were analysed with the Stat View 4.02 statistical software package (Abacus Concepts, Berkley, CA, USA) using the Wilcoxon matched-pairs test, Fisher's exact probability test and Pearson's correlation coefficient. Differences were considered significant when $P$ was <0.05.

### RESULTS

### Autophosphorylation of renaturable protein kinases in colorectal cancer and normal tissue samples

Using the denaturation–renaturation method of proteins separated by SDS-PAGE and electroblotted to Immobilon-P membrane, we identified at least four renaturable kinases (50, 55, 85 and 200 kDa) from both adjacent colon mucosa and adenocarcinoma that exhibit autokinase activity. To prove that radioactivity on the blots represents autophosphorylation of the proteins, several experiments were performed in the pilot studies. Autophosphorylation was obtained using only [$\gamma^{32}$P]ATP, but not [$\alpha^{32}$P]ATP, the radioactivity signals were progressively reduced by increasing concentrations of cold ATP and washes with 1 M potassium hydroxide did not reduce the $^{32}$P-labelled protein bands. Moreover, phosphoamino acid analysis revealed that the 85-kDa kinase autophosphorylates on serine and threonine residues (Rachie et al. 1993). Figure 1 illustrates the typical pattern of autophosphorylation activities in the blotted proteins extracted from four different colorectal cancer tissues and their matched normal mucosa.

In most of the cancer tissue homogenates, 85-kDa kinase exhibited a higher level of autophosphorylation than those in normal matched tissue, although the level of autophosphorylation varied from sample to sample (Figure 2A). The autophosphorylation level in 30 cancer samples ranged from 80 to 1284 arbitrary units with a mean (±s.d.) of 420 ± 303 and was significantly higher ($P < 0.001$) than that of matched normal colorectal mucosa, where the levels ranged from 11 to 766 arbitrary units with a mean (±s.d.) of 192±165. Moreover, in most homogenates from cancer tissue 85-kDa kinase exhibited faster mobility in SDS-PAGE, even when 85-kDa autophosphorylation signals in cancer and matched normal tissue were of the same intensity (Figure 3). The reason for the faster electrophoretic mobility of the 85-kDa kinase-derived form cancer is not known.

In human cancer and adjacent colorectal mucosa homogenates, another prominent renaturable autophosphorylation activity was found around 50 kDa. The level of 50 kDa kinase autophosphorylation activity in cancer homogenates ranged from 141 to 1100 arbitrary units with a mean (±s.d.) of 437 ± 247. But, in contrast to the 85-kDa kinase, the 50-kDa autokinase activity in cancer was significantly lower ($P < 0.05$) than that in normal tissue homogenates, where it ranged from 160 to 1644 arbitrary units with a mean (±s.d.) of 598 ± 416 ($P < 0.05$; Figure 2B).

### Activation of MAP kinase (K) in human colorectal cancers

The MAPK activity in colorectal cancer homogenates ranged from 616 to 8498 c.p.m. with a mean (±s.d.) of 2261 ± 1821, which was significantly higher ($P < 0.001$) than the activity in adjacent mucosa homogenates, where it ranged from 342 to 1894 c.p.m. with a mean (±s.d.) of 913 ± 354. In 14 out of 30 pairs of cancer–normal tissues, MAPK activity in the tumour sample was at least twice as high as that in adjacent mucosa. However, the same variability in MAPK activity was observed (Figure 2C) as in the autophosphorylation activities of 85- and 50-kDa kinases.

### Immunoprecipitation of 85-kDa with anti-RING3 antibodies

As shown in Figure 4, 85-kDa kinase was immunoprecipitated by the immune but not the preimmune anti-RING3 kinase antibodies. These results have proved our earlier observations (Ostrowski et al. 1998), suggesting that 85-kDa kinase is either the same as or related to RING3 kinase.

### Correlation between 85-kDa and MAPK activities with stage of colorectal cancer

To determine whether the tested kinases could be used as prognostic indicators for patients with colorectal cancer, we searched for correlation between 85-kDa and MAPK activities with conventional clinical and pathological parameters.

No correlation was found between 85-kDa autophosphorylation activity and activity of MAPK and patient's age, the site of the disease or tumour size.

 © Cancer Research Campaign 1998

**Figure 4** Immunoprecipitation of 85-kDa. Proteins from colon cancer and adjacent mucosa were incubated with preimmune or immune anti-RING3 chicken antibodies and, following binding to agarose beads bearing anti-chicken IgY antibodies, the immunoprecipitated proteins were assayed as described under Methods

When patients were divided according to a modified Dukes' staging system (Astler et al, 1954), in 12 patients with stage B2, MAPK activity in cancer tissues compared with normal matched mucosa increased by an average of 258±117% and was similar to 18 patients with stage C1 or C2 in whom MAPK activity in cancer tissue increased by an average of 259±203% above the level in adjacent mucosa.

85-kDa autokinase activity examined in cancer tissue from patients with stage B2 increased by an average of 244±131% above the activity in adjacent mucosa and did not differ significantly ($P>0.05$) from those with stage C1 or C2, where 85-kDa autokinase activity in cancer tissue increased by an average of 400±365%.

50-kDa autokinase activity in cancer tissue from patients with stage B2 and C1 or C2 decreased below autokinase activity in adjacent mucosa by an average of 81±34% and 97±54% respectively ($P > 0.05$).

Thus, we were also unable to demonstrate any correlation between MAPK activity or renaturable kinase autophosphorylation activity with conventional prognostic indicators. Consequently, as the pathological disease staging following surgical treatment is the most potent prognostic factor of relapse in colorectal cancer patients, it seems that neither MAPK nor renaturable 85- and 50-kDa kinases can serve as predictors of prognosis for identifying patients with an increased risk of early relapse.

### DISCUSSION

Growth factor-responsive protein kinases are thought to be involved in neoplastic processes. This notion is largely based on studies carried out in cultured cells and the observation that tyrosine kinases can emerge as oncogenes (Rodrigues et al, 1994; Cance et al, 1995). Surprisingly, very few studies have examined kinase activities in intact organs or cancer tissues. Adenocarcinoma of the colon is one of the most common malignancies today (Boring et al, 1991) but very little is known about the role of protein kinases in this malignant disease. The only protein kinase studies in adenocarcinoma of the colon that have been carried out to date examined activity of PKC, which was found to be decreased in this tumour compared with normal tissue (Kopp et al, 1991; Kusunoki et al, 1992; Pongacz et al, 1995). Because of the small amount of information that exists about kinases in the adenocarcinoma of the colon, in this study we chose to examine activities of MAP kinase and renaturable kinases in this tumour.

Based primarily on studies performed in cultured cells, the MAPK cascade is thought to be involved in growth factor-induced cell proliferation (Seger et al, 1995). As such, this cascade might also be involved in the pathogenesis of malignant processes. Growth factor-triggered activation of MAPK is achieved through the engagement of Ras and a serial activation of Raf kinase and MEK. Activation of MEK allows this enzyme to phosphorylate and activate MAPK. Activated MAPK has many targets, both in the cytoplasm and the nucleus, including several transcription factors that presumably mediate the MAPK-mediated mitogenic response. Here we have demonstrated that in nearly each one of the 30 patients MAPK activity was higher in homogenates prepared from colon cancer tissue than the normal adjacent mucosa. This observation is in agreement with other studies in which MAPK activity was shown to be higher in human renal cell carcinoma (Oka et al, 1995), prostate cancer (Magi-Galluzzi et al, 1997) and a subset of acute, but not chronic myelogenous, leukaemia (Towatari et al, 1997). The increased constitutive activity of MAPK found in adenocarcinoma of the colon (Figure 2) and other cancers (Magi-Galluzzi et al, 1997; Towatari et al, 1997), in conjunction with cell culture studies implicating MAPK in the mitogenic responses (Seger et al, 1995), may reflect a role of the MAPK cascade in the generation of the malignant process.

The 85-kDa renaturable kinase is a newly identified enzyme that in some cell types is exclusively nuclear (Denis et al, 1996). The 85-kDa kinase is activated in response to treatment of cells with a myriad of growth factors and cytokines. For example, the 85-kDa autokinase activity in the nucleus is increased after treatment of the EL-4 thymoma cells or 70Z/3 the pre-B cells with IL-1$\alpha$, LPS and IFN-$\gamma$ (Rachie et al, 1993). Systemic administration of EGF, PMA or IL-1$\beta$ into mice increases the autophosphorylation activity of the 85-kDa kinase in multiple organs, including lung, kidney, liver and heart (Ostrowski et al, 1998). Importantly, the 85-kDa autokinase activity is extremely high in leucocytes of patients with various types of chronic and acute leukaemia. In one patient tested the 85-kDa autokinase activity was greatly diminished following remission of acute leukaemia.

Recently, a cDNA encoding an 83-kDa renaturable, serine/threonine kinase (RING3) was characterized in HeLa cells. RING3 kinase was found to be stimulated by the treatment of cell cultures with a number of agents, including IL-1, serum, mitogenic lectins (Denis et al, 1996). In HeLa cells RING3 kinase is exclusively nuclear, and, like 85-kDa kinase, it reveals autophosphorylation activity after protein denaturation and renaturation on the membrane. Anti-RING3 antibodies immunoprecipitated an 85-kDa autokinase activity from colorectal cancer and adjacent mucosa as well as from the mice lung and brain (Ostrowski et al, 1998), suggesting that 85-kDa and RING3 kinases are the same or related enzymes.

Our studies showed that in nearly each one of the 30 patients the 85-kDa autokinase activity was higher than in the matched normal tissue. However, the most consistent observation was the faster electrophoretic mobility of the 85-kDa kinase derived from the tumours. Both of the observations, therefore, demonstrate that this enzyme is altered in adenocarcinoma of the colon. Because this kinase is responsive to growth factors (Rachie et al, 1993; Ostrowski et al, 1998), is extremely active in leukaemic cells (Denis et al, 1996; our unpublished observations) and is constitutively activated in colon cancer (Figures 1 and 3), it may be involved in the neoplastic processes.

We also found that the autophosphorylation activity of one of the renaturable kinases, 50-kDa kinase, in colon cancer was lower than in the normal adjacent tissue. The significance of this obser-

© Cancer Research Campaign 1998

vation is not clear but is similar to the observation that PKC activity is lower in colorectal adenomas and adenocarcinomas (Kopp et al, 1991; Kusunoki et al, 1992; Pongacz et al, 1995), as well as in liver cancer compared with normal tissue in those organs (Chang et al, 1996).

In summary, this study demonstrates that the constitutive activities of MAPK and autophosphorylation activity of 85-kDa kinase are increased, whereas the autophosphorylation activity of another kinase, 50 kDa, is decreased in adenocarcinoma of the colon. These observations suggest that the signal transduction processes in the neoplastic cells are greatly altered, although none of the studied kinase activities could serve as prognostic indicators. Whether the altered activity of MAPK, 85- and 50-kDa kinases reflects involvement of these enzymes in the neoplastic process or whether they represent associated phenomena is a key issue that needs to be explored in order to better understand the pathogenesis of this and other cancers.

### ACKNOWLEDGEMENTS

This work was supported by grants from the Polish Committee for Scientific Research (KBN 4 P05A 114 10 to JO and NIH GM42508 and GM455134 to KB).

### REFERENCES

Astler VB and Coller FA (1954) The prognostic significance of direct extension of the carcinoma of the colon and rectum. *Ann Surg* **139**: 846–859

Boring CC, Squires TS and Tong T (1991) Cancer statistics. *CA Cancer J Clin* **41**: 19–36

Cance WG and Liu ET (1995) Protein kinases in human breast cancer. *Breast Cancer Res Treat* **35**: 105–114

Chang K-J, Lin J-K, Lee P-H, Hsieh Y-S, Cheng C-K and Lin J-Y (1996) The altered activity of membrane-bound protein kinase C in human liver cancer. *Cancer Lett* **105**: 211–215

Denhardt DT (1996) Signal-transduction protein phosphorylation cascades mediated by Ras/Rho proteins in the mammalian cell: the potential for multiplex signaling. *Biochem J* **318**: 729–747

Denis GV and Green MR (1996) A novel, mitogen-activated nuclear kinase is related to a *Drosophila* developmental regulator. *Genes Dev* **10**: 261–271

Hen J, Jiang Y, Li Z, Kravchenko VV and Uleritch RJ (1997) Activation of the transcription factor for MEF2C by MAP kinase p38 in inflammation. *Nature* **386**: 296–299

Hills CS and Treisman R (1995) Transcriptional regulation by extracellular signals: mechanism and specificity. *Cell* **80**: 199–211

Hunter T (1995) Protein kinases and phosphatases: the Yin and Yang of protein phosphorylation and signaling. *Cell* **80**: 225–236

Kopp R, Noelke B, Santer G, Schileberg FW, Panmagartner G and Pfeiffer A (1991) Altered protein kinase C activity in human colonic adenoma and carcinomas. *Cancer Res* **51**: 205–210

Kusunoki M, Sakanone Y, Hatada T, Yanagi F, Tamamura T and Utsunomiya J (1992) Protein kinase C activity in human colonic adenoma and colorectal carcinoma. *Cancer* **69**: 24–30

Levitzki A (1996) Targeting signal transduction for disease therapy. *Curr Opin Cell Biol* **8**: 239–242

Madden K, Shen YJ, Backz K, Andrews B and Snydr M (1997) SBF cell regulator as a target of the yeast PKC-MAP kinase pathway. *Science* **275**: 1781–1784

Magi-Galluzzi C, Mishra R, Fiorentino M, Montironi R, Yoa H, Capodieci P, Wishnow K, Kaplan I, Stork PJS and Loda M (1997) Mitogen-activated protein kinases phosphatase 1 is overexpressed in prostate cancers and is inversely related to apoptosis. *Lab Invest* **76**: 37–51

Marois R, Wynne J and Treisman R (1993) The SRF accessory protein ELK-1 contains a growth factor-regulated transcriptional activation domain. *Cell* **73**: 381–393

Marshall CJ (1994) MAP kinase kinase kinase, MAP kinase kinase and MAP kinase. *Curr Opin Genes Dev* **4**: 82–89

Nishida E and Gotoh Y (1993) The MAP kinase cascade is essential for diverse signal transduction pathways. *Trends Biochem Sci* **18**: 128–131

Oka H, Chatani Y, Hoshino R, Ogawa O, Kakehi Y, Terachi T, Okada Y, Kawaichi M, Kohno M and Yoshida O (1995) Constitutive activation of mitogen-activated protein (MAP) kinases in human renal cell carcinoma. *Cancer Res* **55**: 4182–4187

Ostrowski J, Florio SK, Denis GV, Suzuki H and Bomsztyk K (1998) Stimulation of p85/RING3 kinase in multiple organs after systematic administration of mitogens into mice. *Oncogene* **16**: 1223–1227

Pelech SL and Sanghera JS (1992) Mitogen-activated protein kinases: versatile transducers for cell signaling. *Trends Biochem Sci* **17**: 233–238

Pongacz J, Clark P, Neoptolomos JP and Lord JM (1995) Expression of protein kinase C isoenzymes in colorectal cancer tissue and their differential activation by different bile acids. *Int J Cancer* **61**: 35–39

Pulverer BJ, Kyriallis JM, Avruch J, Nikolakaki E and Woodgett JR (1991) Phosphorylation of c-jun mediated by MAP kinase. *Nature* **353**: 670–674

Rachie NA, Seger R, Valentine MA, Ostrowski J and Bomsztyk K (1993) Identification of an inducible 85-kDa nuclear protein kinase. *J Biol Chem* **268**: 22143–22149

Reuter CWH, Catling AD and Weber MJ (1995) Immune complex kinase assays for mitogen-activated protein kinase and MEK. *Methods Enzymol* **225**: 245–256

Rodrigues GA and Park M (1994) Oncogenic activation of tyrosine kinases. *Curr Opin Genet Dev* **4**: 15–24

Seger R and Krebs EG (1995) The MAPK signaling cascade. *FASEB J* **9**: 726–735

Towatari M, Lida H, Tanimoto M, Iwata H, Hamaguchi M and Saito H (1997) Constitutive activation of mitogen-activated protein kinase pathway in acute leukemia cells. *Leukemia* **11**: 479–484

Treisman R (1996) Regulation of transcription by MAP kinase cascades. *Curr Opin Cell Biol* **8**: 205–215

Whitmarsh AJ and Davis RJ (1996) Transcription factor AP-1 regulation by mitogen-activated protein kinase signal transduction pathways. *J Mol Med* **74**: 589–607

© Cancer Research Campaign 1998